# Seven cases of robot-assisted radical cystectomy with Bordeaux neobladder: A clinical observation

**DOI:** 10.14440/bladder.0049

**Published:** 2025-08-27

**Authors:** Hua Yang, Xuchang Liu, Bo Wu, Jingjing Li, Yangjie Gao, Guochen Zhao, Wenjie Yang, Junyuan Bing, Xiaoming Cao

**Affiliations:** Department of Urology, First Hospital of Shanxi Medical University, Taiyuan, Shanxi 030001, China

**Keywords:** Robot-assisted radical cystectomy, Bordeaux neobladder, Bladder cancer, Clinical observation

## Abstract

**Background:**

Bladder cancer (BCa) poses a significant global health burden with mounting incidence and mortality rates. Effective treatment strategies are urgently needed to alleviate the disease burden and improve patients’ quality of life (QoL).

**Objective:**

This study aimed to evaluate the efficacy of robot-assisted radical cystectomy (RARC) plus the Bordeaux robotic intracorporeal orthotopic neobladder (riONB) technique for the treatment of BCa.

**Methods:**

We retrospectively analyzed clinical data from seven male patients who had undergone RARC with Bordeaux riONB between June 2023 and November 2024. The median age was 68 years, and the median body mass index was 24.2 kg/m^2^. The study assessed their demographic data, intra- and post-operative parameters, pathological characteristics, and complications.

**Results:**

The median operation time lasted for 583 min, with a median estimated blood loss of 375 mL and a median post-operative hospital stay of 10 days. Pathological results showed that five patients (71.4%) were at stage ≥T2. According to the Clavien-Dindo classification, six patients (85.7%) had grade I – II complications. At 6 months post-surgery, the average maximum cystometric capacity measured 397 mL, the average post-void residual urine volume was 29 mL, the average urine flow rate was 5.6 mL/s, and the incontinence-QoL score was 85. Daytime urinary control was good in five patients (71.4%), and nighttime control was good in three patients (42.9%).

**Conclusion:**

The results indicate that RARC + Bordeaux riONB is a safe and effective surgical option for BCa, accomplishing good patient recovery and significant clinical efficacy. However, the study was subject to limitations of a small sample size and a short follow-up period. Further research with larger cohorts and longer follow-up is warranted to confirm these findings.

## 1. Introduction

According to GLOBOCAN 2020, there were approximately 573,000 new cases of bladder cancer (BCa) and 213,000 deaths from BCa in 2020.^1^ The incidence and mortality rates of BCa have shown a consistent upward trend over the years. By 2040, the annual incidence and mortality rates of BCa are projected to rise by 72.8% and 86.6%, respectively.^2^ These projections underscore the pressing need for effective strategies to ease the disease burden of BCa and improve the quality of life (QoL) of the patients.

In recent years, the research endeavors on bladder replacement surgery have focused on two technological approaches: (i) Exploring the clinical feasibility of tissue-engineered bladders; and (ii) systematically improving robot-assisted entirely intracorporeal orthotopic neobladders (ONBs). Tissue-engineered bladders aim to replicate the complex functions of the bladder, including urine storage and voiding, while minimizing complications associated with present surgical practices. The development of biomaterials, including cell-free matrices, hydrogels, and nanomaterials, along with emerging tissue engineering technologies, such as cell therapy and organoids for bladder regeneration and functional recovery, has shown potential in creating functional bladder substitutes.^3^-^5^ However, this approach continues to face significant challenges. The autonomous regeneration of complex tissues in the human body remains a major hurdle in regenerative medicine. Furthermore, due to technological immaturity and staggering costs, widespread application in the near term remains unlikely.

Robot-assisted radical cystectomy (RARC), combined with robotic intracorporeal ONB (riONB), represents a contemporary surgical technique that uses the patient’s intestinal tissue to reconstruct the bladder after robot-assisted cystectomy.^6^ This technology has shown significant advantages in improving patients’ QoL, especially in avoiding stoma and related complications. It is one of the mainstream treatments for locally resectable muscle-invasive BCa (MIBC) without distant metastasis and high-risk non-MIBC (NMIBC).^7^

Robot surgery has become incrementally safe and mature. At present, different types of ileal neobladder procedures have been clinically applied, and their common goals include minimal urine storage and reabsorption, good compliance, low pressure and high capacity, no reflux, no urinary incontinence, and complete emptying.^8^-^12^ The reconstruction of the urine storage and urination function of the new bladder is the main challenge confronting this technology. In 2016, Asimakopoulos *et al*.^13^ innovatively proposed the modified Y-shaped orthotopic (Bordeaux) neobladder. They built a virtually spherical new bladder through a small segment of the ileum, keeping the ureter aligned in a natural position. The new bladder had a good average capacity, no residual urine post-voiding, no need for catheterization, and a low complication rate, resulting in good and safe functional outcomes. The Bordeaux neobladder, with its core advantages of a rapidly formed spherical shape and low-pressure urine storage, serves as a “bridge” option between present standard neobladder procedures and future bioengineered bladder alternatives. On the basis of the subsequent research by Asimakopoulos *et al.*,^14^ our center began to perform RARC + Bordeaux riONB surgery in 2023. This study aimed to evaluate the efficacy of RARC + Bordeaux riONB, including surgical data and post-operative follow-up data.

## 2. Materials and methods

### 2.1. Patients

This study retrospectively analyzed clinical data from patients who had undergone RARC in combination with the Bordeaux riONB procedure at our institution between June 2023 and November 2024. Before surgery, all patients had received standard urinary system computer tomography or magnetic resonance imaging scans and were confirmed to have BCa through cystoscopic biopsy or diagnostic transurethral resection. All patients were clinically and pathologically staged according to the 8^th^ edition of the American Joint Committee on Cancer Tumor-Node-Metastasis system. This cohort included patients with pathological tumor stage (pT) < 3b, cN0 – 1, M0, and negative intraoperative frozen section margins at the distal urethra and bladder neck, thereby meeting the European Association of Urology (EAU) guideline safety criteria for orthotopic bladder substitution.^15^ At present, no study specifies the applicable pathological stages for the Bordeaux neobladder. Based on EAU guidelines, the absolute contraindications for ONBs are positive surgical margins at the distal urethra, bladder neck/urethral carcinoma *in situ*, or anywhere on the specimen, and, in men, extensive prostatic involvement. The tumor stage alone is not a contraindication. For ≥pT2 stages, if complete resection with negative margins is possible, ONBs can be considered. A large-scale multicenter clinical dataset shows that, for ≥pT2 (including some pT3) patients, ONBs are non-inferior to ileal conduits and may offer survival benefits after strict staging and lymphadenectomy.^16^ Advancements in neobladder procedures have improved overall bladder-voiding function. Some experts suggest that pT3 – T4a but resectable lesions are increasingly no longer considered absolute contraindications for ONBs, as local recurrence typically does not affect neobladder function.^17^ In this study, patients ≤75 years with good renal function and without significant pelvic fixation or urethral involvement were considered. For individuals with ≥T2 disease, the Bordeaux neobladder can still offer low-pressure, high-capacity function and QoL benefits, provided that multidisciplinary assessment, optimized perioperative risk control, and rigorous long-term follow-up are ensured. The inclusion criteria were as follows: (i) Diagnosis of MIBC or high-risk NMIBC necessitating radical cystectomy; (ii) no evidence of extensive retroperitoneal lymph node involvement or distant metastasis on imaging; (iii) absence of severe hepatic or renal dysfunction, cardiopulmonary issues, intestinal damage, or other contraindications to surgery; (iv) no tumor involvement of the urethra and/or prostate; (v) having obtained patient consent to undergo the Bordeaux orthotopic ileal neobladder surgery, with full informed consent and pre-operative functional assessment; and (vi) availability of complete follow-up data. The exclusion criteria included: (i) Severe pelvic adhesions or other conditions precluding robotic surgery; (ii) extensive interoperative tumor spread or inability to complete the ONB surgery; (iii) loss to follow-up or incomplete clinical data. The final study cohort comprised seven male patients.

### 2.2. Surgical technique

**Figure 1:**
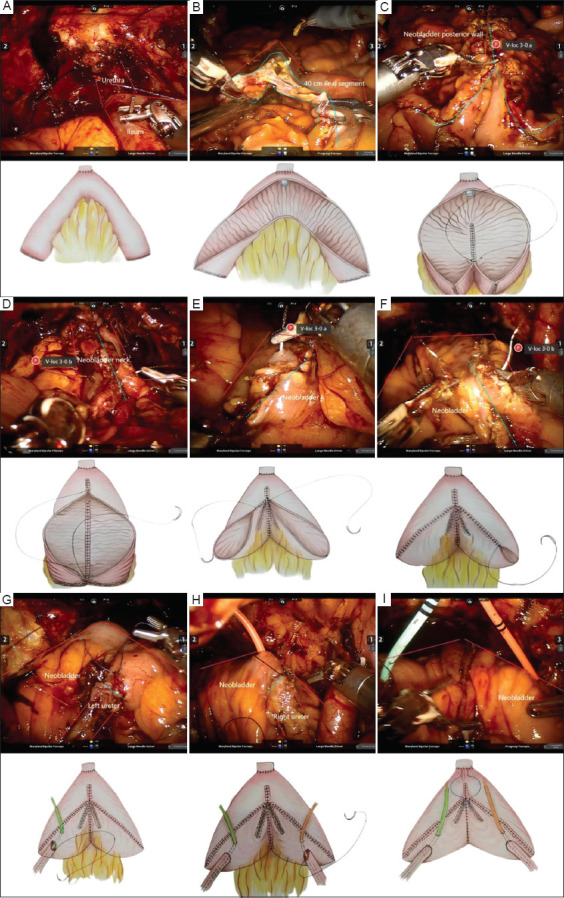
Surgical procedure of robot-assisted radical cystectomy, combined with robotic intracorporeal orthotopic neobladder. (A) At 15 cm away from the ileocecal junction, a 40 cm segment of ileum was selected, and the midpoint was transversely cut open about 2 – 3 cm using scissors, then a V-loc 3-0 suture was used for anastomosis with the urethra. (B) The 40 cm ileal segment was excised, intestinal continuity was restored, and this part of the ileum was detubularized. (C) The posterior wall of the new bladder was sutured first with a V-loc 3-0 suture. (D) Four to six stitches were used with a V-loc 3-0 suture to reconstruct the bladder neck. (E) The left half of the anterior wall of the new bladder was sutured with a V-loc 3-0 suture. (F) The right half of the anterior wall of the new bladder was sutured with a V-loc 3-0 suture. (G) The left ureter was anastomosed to the new bladder. (H) The right ureter was anastomosed to the new bladder. (I) Two single J-stents were brought out through the pubic bone for drainage, keeping the new bladder decompressed.

First, the surgeon selected a 40 cm segment of the ileum, located 15 cm proximal to the ileocecal junction, and made a 2 – 3 cm transverse incision at the midpoint. A V-loc 3-0 suture (Medtronic, Ireland) was then used to perform an anastomosis between the urethra and the ileum. Following the resection of the 40 cm ileal segment, intestinal continuity was re-established, and the ileal segment was detubularized. A V-loc 3-0 suture was used to suture the posterior wall of the newly constructed bladder, followed by another V-loc 3-0 suture to place four to six continuous stitches to reconstruct the bladder neck. The left and right halves of the anterior wall of the new bladder were then sutured separately. Finally, the ureters were anastomosed to the new bladder, and a single J-stent was inserted percutaneously for drainage to maintain the new bladder in a decompressed state (Figure 1).

### 2.3. Outcome measures

Patient demographics, intraoperative and post-operative parameters, pathological characteristics, and complications were retrospectively evaluated. Demographic data included age, gender, clinical stage, history of transurethral resection of bladder tumor (TUR-Bt), history of neoadjuvant therapy, and American Society of Anesthesiologists (ASA) classification; intraoperative data involved operation time (in minutes) and intraoperative blood loss (in milliliters); post-operative data covered post-operative hospital stay (in days), surgical margin status, number of positive lymph nodes, post-operative pathological stage, related complications, and functional recovery. Post-operative follow-up was conducted at 3, 6, 9, and 12 months after surgery. Patients completed a full set of urodynamic examinations 6 – 12 months post-operatively, and some patients underwent computed tomography urography (CTU) 12 months after the operation. Urinary tract reconstruction was performed, and the following parameters were recorded: Maximum cystometric capacity (MCC, mL), post-void residual urine volume (PVR, mL), average urine flow rate (Q_ave_, mL/s), maximum urine flow rate (Q_max_, mL/s), maximum urine flow rate time (TQ_max_, s), urine flow time (FT, s), and bladder compliance. Imaging was used to evaluate the structure and changes of the upper urinary tract. The subjective urinary control ability and urination habits of the patients were assessed through telephone interviews and outpatient consultations. Urinary control ability was categorized into daytime and nighttime control according to the standards of the International Continence Society, strictly defined as follows: If the patient is completely normal and does not require any protective measures, it is “good” (with control); if a maximum of one urine pad is needed during the day or night, it is “satisfactory” (socially controlled); if more than one urine pad is needed during the day or night, it is “unsatisfactory” (no control).^18^ The incontinence-QoL scale was used to evaluate the impact of urinary incontinence on the patient’s QoL. Renal pelvic dilatation was classified into mild hydronephrosis (1.0 – 2.0 cm), moderate hydronephrosis (2.1 – 3.5 cm), and severe hydronephrosis (>3.5 cm) in terms of the degree of renal pelvic dilatation.

### 2.4. Statistical analysis

Statistical analysis was performed by using (Statistical Package for Social Sciences 22.0, IBM, United States). Continuous variables that followed a normal distribution pattern are presented as means, while those that do not follow a normal distribution are expressed as medians. Categorical variables are reported as case numbers and percentages.

## 3. Results

A total of seven male patients were included in this study, all having undergone RARC combined with Bordeaux riONB. The median age of the patients was 68 years (range: 62 – 74 years), and the median body mass index (BMI) was 24.2 kg/m^2^ (range: 21.5 – 26.7 kg/m^2^). Pathologically, five patients (71.4%) were staged ≥T2, and two patients (28.6%) were <T2. Three patients (42.9%) received neoadjuvant chemotherapy, and four patients (57.1%) had a history of TUR-Bt. On the ASA scale, five patients (71.4%) were graded I – II, and two patients (28.6%) were rated III – IV (Table 1).

**Table 1:** Pre-operative data of the seven patients

Variables	Values
Age (year), median (range)	68 (62 – 74)
BMI (kg/m^2^), median (range)	24.2 (21.5 – 26.7)
pT stage, *n* (%)	
≥T2	5 (71.4)
<T2	2 (28.6)
Neoadjuvant chemotherapy, n (%)	3 (42.9)
History of transurethral resection of bladder tumor, n (%)	4 (57.1)
ASA score, *n* (%)	
I – II	5 (71.4)
III – IV	2 (28.6)

Abbreviations: ASA: American Society of Anesthesiologists; BMI: Body mass index.

The median operation time lasted 583 min (range: 475 – 728 min), the median estimated blood loss was 375 mL (range: 150 – 600 mL), and the median post-operative hospital stay was 10 days (range: 8 – 13 days). Regarding positive surgical margins, no cases had positive ureteral margins or positive lymph nodes, but one case (14.3%) had positive soft tissue margins. According to the Clavien-Dindo classification, six patients (85.7%) had grade I – II complications, and one patient (14.3%) had grade III – V complications (Table 2).

**Table 2:** Intraoperative and post-operative data of the seven patients

Variables	Values
Operative time (min), median (range)	583 (475 – 728)
Estimated blood loss (mL), median (range)	375 (150 – 600)
Hospitalization time (day), median (range)	10 (8 – 13)
Positive margins, *n* (%)	
Ureteral margins	0 (0)
Soft-tissue margins	1 (14.3)
Lymph node positive margins, *n* (%)	0 (0)
pT stage, *n* (%)	
≥T2	5 (71.4)
<T2	2 (28.6)
Clavien-Dindo complication score, *n* (%)	
Grade I – II	6 (85.7)
Grade III – V	1 (14.3)

The urinary control results at 6 months after surgery indicated that the average MCC was 397 mL (range: 379 – 412 mL), the average PVR was 29 mL (range: 7 – 40 mL), the Q_ave_ was 5.6 mL/s (range: 2.3 – 8.2 mL/s), the Q_max_ was 12.9 mL/s (range: 8.6 – 15.9 mL/s), the TQ_max_ was 22.5 s (range: 3.9 – 44.8 s), and the FT was 33.3 s (range: 10.6 – 42.1 s). In terms of daytime urinary control, five patients (71.4%) did not require the use of urine pads, two patients (28.6%) used one urine pad, and no patient used more than one urine pad. In terms of nighttime urinary control, three patients (42.9%) did not require the use of urine pads, three patients (42.9%) used one urine pad, and one patient (14.3%) used more than one urine pad. The incontinence-QoL score was 85 (ranged 80 – 90). In terms of hydronephrosis, two patients (28.6%) had mild hydronephrosis, and one patient (14.3%) had moderate hydronephrosis (Table 3).

**Table 3:** Post-operative urinary continence data of the seven patients

Variables	Values
MCC (mL), mean (range)	397 (379 – 412)
PVR (mL), mean (range)	29 (7 – 40)
Q_ave_ (mL/s), mean (range)	5.6 (2.3 – 8.2)
Q_max_ (mL/s), mean (range)	12.9 (8.6 – 15.9)
TQ_max_ (s), mean (range)	22.5 (3.9 – 44.8)
FT (s), mean (range)	33.3 (10.6 – 42.1)
Daytime continence, *n* (%)	
0 Pad (good)	5 (71.4)
1 Pad (satisfactory)	2 (28.6)
≥1 Pad (unsatisfactory)	0 (0)
Nighttime continence, *n* (%)	
0 Pad (good)	3 (42.9)
1 Pad (satisfactory)	3 (42.9)
≥ 1 Pad (unsatisfactory)	1 (14.3)
Incontinence-QoL score, mean (range)	85 (80 – 90)
Hydronephrosis, *n* (%)	
Mild	2 (28.6)
Moderate	1 (14.3)
Severe	0 (0)

Abbreviations: FT: Flow time; MCC: Maximum cystometric capacity; PVR: Post-void residual of urine; Q_ave_: Average urine flow rate; Q_max_: Maximum urine flow rate; TQ_max_: Maximum urine flow rate time.

**Figure 2:**
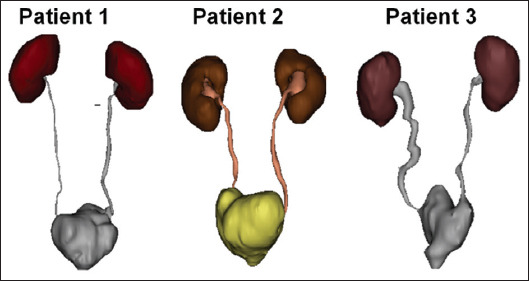
Computed tomography urography imaging of three patients, 12 months post-surgery

These results demonstrated the safety and reliability of the surgical technique presented in this study, along with favorable patient recovery and significant clinical efficacy. All seven patients were followed up for more than 6 months after surgery, and no tumor recurrence or metastasis has been observed to date. CTU imaging of three patients 12 months after surgery revealed well-preserved morphology of both upper urinary tracts and new bladders (Figure 2).

## 4. Discussion

At present, RARC plus riONB is extensively utilized in the treatment of BCa, but it also requires strict evaluation of the patient’s basic conditions before surgery. Patient selection is closely related to the success of the surgery and the occurrence of post-operative complications.^19^,^20^ A previous study has found that patients undergoing ONB with a BMI of ≥27 kg/m^2^ were at significantly increased risk of retention and needed self-catheterization.^21^ Another study showed that patients with BMI ≥25 kg/m^2^ who received RARC plus riONB experienced greater blood loss and longer operation time. Although the final outcomes indicated that obesity exerted no significant impact on the success of the operation and the occurrence of post-operative complications, intra-abdominal fat may obstruct the operational procedure or increase the risk of complications. Robot-assisted surgeries are generally more technically demanding for surgeons, thereby introducing unpredictable procedural risks.^22^ Patients with higher ASA scores have been reported to have poor long-term QoL,^23^ and an ASA score of ≥3 has been identified as a predictor of complications within 90 days after RARC with intracorporeal urinary diversion.^24^

Y-shaped and Bordeaux ONB were priorly reported to have the shortest operation time across all other techniques (304.7 and 315.0 min, respectively), with an average operation time of approximately 400 min. This finding may be explained in part by the relatively simpler configuration and folding procedures required for Y-shaped and Bordeaux ONB compared to other neobladder techniques.^25^ It is worth noting that shortening the operation time not only helps improve the efficiency of the operation but may also have a positive impact on the patient’s post-operative recovery. However, the choice of specific technology still needs to be comprehensively considered based on the patient’s specific situation and the experience of the surgical team.

After RARC plus Bordeaux riONB operation, assessing urinary function recovery through urodynamic testing is an important part of the patients’ recovery process, as it provides crucial physiological data. In a cohort study, 28 patients who underwent riONB were compared with 79 patients who received open ONB, and the results showed that the volume and pressure of the neobladder created by the fully robotic-assisted laparoscopic technique were acceptable.^26^ Unfortunately, currently no standard is available for urodynamic testing of ONB, and the evaluation of the neobladder’s storage and voiding functions mostly relies on the results of conventional urodynamic tests. The assessment of specific therapeutic effects often requires comparison with the results reported by other researchers. Grobet-Jeandin *et al*.^27^ conducted a study involving 14 patients who underwent RARC with a Y-shaped neobladder. These patients exhibited a median maximal cystometric capacity of 495 mL (interquartile range [IQR]: 410 – 606 mL) and a median compliance of 35.5 mL/cm H_2_O (IQR: 28 – 62 mL/cm H_2_O) 6 months after operation. Except for three patients (22%) who required clean intermittent self-catheterization, PVR was <30 mL in all participants. Asimakopoulos *et al*.^14^ revealed that, on average, 27 months post-operatively, the mean MCC of the neobladder was 431 cm^3^ (range: 200 – 553 cm^3^). The mean PVR volume was 101.6 mL (range: 0 – 310 mL), with a rate of clean intermittent catheterization required being at 17.6%. In our study, urinary control measures of 6 months post-surgery showed an average MCC of 397 mL, average PVR of 29 mL, average Q_ave_ of 5.6 mL/s, average Q_max_ of 12.9 m/s, average TQ_max_ of 22.5 s, and average FT of 33.3 s. Six months after surgery, the patients’ MCC and PVR were close to the international normal range, indicating that the urine storage function was good and no clean intermittent catheterization was required.^28^,^29^ However, urodynamic indicators (Q_ave_, Q_max_, TQ_max_, and FT) generally deviated from normal standards. The reason lies in that the neobladder possesses neither sensory function nor active contraction ability. When urinating, the abdominal muscles need to be contracted to increase abdominal pressure to compress the neobladder, and the external urethral sphincter has to be relaxed to complete the urination process. Therefore, patients require long-term urination training.

A study by Zhou *et al*.^30^ showed that, 6 months after Hautmann ONB surgery, 73.3% of patients had good, 16.7% had satisfactory, and 6.7% had unsatisfactory daytime continence. Nighttime continence of the patients was good in 16.7%, satisfactory in 60.0%, and unsatisfactory in 20.0%. By 12 months, 75.0% of the patients reported good, 15.0% reported satisfactory, and 5.0% reported unsatisfactory daytime continence. Nighttime continence of the patients improved to good in 15.0%, to satisfactory in 65.0% satisfactory, and was unsatisfactory in 15.0%. In our series, 6 months post-operation, 71.4% of patients had good daytime continence, while 42.9% had good nighttime continence. Daytime continence was superior to nighttime continence, and we infer that one explanation for this finding is the involuntary contractions of the neobladder. The intestinal segment that constitutes the new bladder has the physiological characteristic of rhythmic peristalsis, which leads to involuntary contractions of the new bladder. Although detubularization destroys the continuous circular muscle layer of the intestine, it cannot completely prevent involuntary contractions of the neobladder. In the state of relaxation of the external urethral sphincter at night, the involuntary contractions of the neobladder worsen the outcome of urinary incontinence.^31^ Alberto *et al*.^32^ reported the urinary continence outcomes at 12 months post-RARC with ONB (including techniques, such as Studer/Wiklund, S pouch, Gaston, vescica ileale Padovana, or Hautmann technique), with daytime and nighttime urinary continence rates being at 86% and 66%, respectively, and the continence outcomes were unrelated to the type of neobladder. Unlike the Studer or Hautmann neobladders, which may require more complex anastomoses and a longer learning curve, the Bordeaux approach simplifies bladder reconstruction while preserving functional outcomes and reducing surgical complexity. A study found that, at 12 months post-ONB surgery, 78.3% of men and 64.0% of women reported social urinary continence (at most 1 safety pad per 24 h), while 27.3% of men and 44.0% of women reported severe incontinence. Interestingly, male gender was identified as an independent predictor of not using pads at 6 months post-surgery.^33^ In our study, we selected only male patients, which may be one of the possible reasons for the good surgical outcomes. These results further support the utility of RARC plus Bordeaux riONB in male patients. Interestingly, the literature reports that the ONB may have a certain positive effect on the recovery of erectile function after surgery due to the preservation of the urethra and possible nerve protection. For example, in one study, male patients who underwent ONB reconstruction were more likely to recover erections after surgery than those receiving ileostomy (6/26 vs. 1/31). However, some studies found no significant difference in sexual function scores between the two groups of patients.^34^ We will include erectile function questionnaires, such as the International Index of Erectile Function-5 in the follow-up to facilitate detailed analysis of this item in future studies.

It should be noted that ONB surgery alters the movement patterns and mechanisms of the intestines and the original bladder. Therefore, urination training is necessary to restore normal micturition.^35^ Perimenis *et al*.^36^ focused on the perioperative and home rehabilitation of patients with ONB and implemented urination-behavior interventions for three and 6 months, including Kegel exercises (10 times/h) that empty the bladder every 2 h during the day, and every 3 h at night. The 5-year follow-up results showed good neobladder function in the subjects. Patients with an ONB can approach a normal storage volume and restore their QoL through scheduled and measured filling and emptying, which also helps them develop a psychological sense of needing to urinate and empty their bladder, forming a urination habit. In our study, this is also an important factor that needs to be considered.

This study has limitations. First, the sample size was only seven cases, and the statistical power was insufficient, which may not be able to detect the real difference. Second, there was no control group, and it was only a single-arm observation, lacking comparative analysis. These design flaws may lead to increased risks of selection bias (such as only including specific patients who satisfied the criteria) and confounding bias (the influence of other uncontrolled factors on the results). Further verification with large samples and control designs is warranted in the future.

## 5. Conclusion

Based on our findings, although the number of cases reported in this study is relatively small, the surgical safety, neobladder function recovery, and urinary control outcomes of RARC plus Bordeaux riONB showed a positive trend.

## Data Availability

The datasets used and analyzed during the present study are available from the corresponding author on reasonable request.
